# Supplementary material for: Collaborative intelligence in AI: Evaluating the performance of a council of AIs on the USMLE
Source: PLOS Digit Health. 2025 Oct 9;4(10):e0000787. doi: 10.1371/journal.pdig.0000787 (PMC12510544; doi:10.1371/journal.pdig.0000787)
Supplement: S2 File — (ZIP) [file pdig.0000787.s002.zip › S2 Data Raw data files containing unprocessed question inputs and LLM outputs including transcripts of deliberations/STEP3_Question-103-1_Type-MC-NJ.docx]

**Facilitator** **[Round 1]:** A 16-year-old girl is brought to the office by her mother because she is concerned that she may have contracted meningitis from her soccer teammate, who was diagnosed with meningococcal meningitis and admitted to the hospital yesterday. The patient's soccer team traveled to a neighboring state to participate in a tournament 1 week ago and she shared a hotel room with the girl who was hospitalized. The patient feels well but is concerned she may have ""caught the same bug."" Medical history is remarkable for asthma. Medications include inhaled albuterol. Vital signs are temperature 37.2°C (98.9°F), pulse 64/min, respirations 16/min, and blood pressure 107/58 mm Hg. Physical examination shows no abnormalities. Which of the following is the most appropriate intervention for this patient at this time? (A) Administer the meningococcal vaccine (B) Prescribe doxycycline (C) Prescribe penicillin (D) Prescribe rifampin (E) Assure the patient that no anti-infective prophylaxis is necessary

**AI_B:** RESTATING THE QUESTION: You are asking about a 16-year-old female who was exposed to meningococcal meningitis via her soccer teammate who has been hospitalized already. The girl is currently symptom-free and her medical history reveals asthma, treated with inhaled albuterol. The query is regarding the most appropriate intervention at this time, with the options being: (A) vaccination against meningococcus, (B) prescribing doxycycline, (C) prescribing penicillin, (D) prescribing rifampin, or (E) assuring the patient that no prophylactic anti-infective treatment is necessary. PLANNING: It's pertinent to assess whether the exposure warrants any chemoprophylactic intervention given a potential risk to develop meningitis after exposure. It is important to differentiate between primary prevention via vaccination and urgent prophylaxis in the case of close contact with confirmed meningococcal disease. Prophylaxis in case of meningococcal exposure usually involves antibiotic treatment rather than vaccination. The vaccine doesn't offer immediate protection; rather, it works over a longer period to stimulate the immune system against future exposure. CLINICAL REASONING: Given that the girl shared a hotel room with a confirmed case of meningococcal meningitis, the level of exposure is high-risk, necessitating chemoprophylaxis. There are several antibiotics used for prophylaxis in such cases: rifampin, ciprofloxacin, and ceftriaxone. Doxycycline is not typically used, and the commencing of penicillin would be for treatment rather than prophylaxis. Assuring the patient that no anti-infective prophylaxis is necessary could potentially be dangerous due to the significant chance for her to contract the disease due to the close contact. CONCLUDING: Considering that the patient had close contact with a confirmed case of meningococcal meningitis, it would be appropriate to initiate a course of prophylactic antibiotics. Vaccination would not provide immediate enough protection, and saying no intervention is necessary would expose the patient to possible health risks. RESPONSE: The most appropriate intervention for this patient at this time would be to (D) Prescribe rifampin, which is an effective chemoprophylactic agent against Neisseria meningitidis, the causative organism of meningococcal meningitis.

**AI_C:** RESTATING THE QUESTION: This query asks about the suitable course of action for a patient who might have been exposed to meningococcal meningitis but is currently asymptomatic. Meningococcal meningitis is an acute bacterial disease caused by Neisseria meningitidis that affects the protective membranes covering the brain and spinal cord. Prophylaxis refers to measures taken to prevent diseases, especially those where the patient has had close, consistent contact with a confirmed case. PLANNING: Assumptions made here include that the girl has been exposed to N. meningitidis, that she might manifest symptoms soon and might therefore require prophylactic treatment, and that the prophylactic treatment to be prescribed should be effective against N. meningitidis. However, an alternate assumption could be that the girl might not have been infected or might have a naturally resilient immune response that could protect her against the disease. This assumption will be evaluated and prioritized based on a clinical approach that considers epidemiology, the incubation period of the disease, her present physical state, and her medical history. CLINICAL REASONING: Given that meningococcal disease can be rapidly fatal, prophylaxis for close contacts of infected individuals is crucial, regardless of the presence or absence of symptoms at the time of exposure. Our patient falls into the category of close contacts as she shared a room with the infected person during a tournament. CONCLUDING: Even though the patient is currently asymptomatic, considering the risk associated with potential exposure to N. meningitidis, it is sensible to start prophylaxis immediately. The Centers for Disease Control and Prevention recommends that close contacts of persons with meningococcal disease should receive prophylactic antibiotics to prevent infection, irrespective of their vaccination status. RESPONSE: Option (D) To prescribe rifampin would be the most appropriate intervention for this patient at this time. Rifampin is commonly used as it effectively eradicates N. meningitidis from the nasopharynx, thereby reducing risk of disease. Other antibiotics used for prophylaxis include ciprofloxacin and ceftriaxone, but these are not listed among the options here.

**AI_E:** (1) RESTATING THE QUESTION: A young female patient with no physical abnormalities and a history of asthma, has had close contact with a recent case of meningococcal meningitis. Her mother is worried that she could have also caught the pathogen, even though she's currently exhibiting no signs of illness. We must ascertain the best preventative response in this scenario. (2) PLANNING: The immediate assumption may be to administer the meningococcal vaccine (option A), however, it does not confer instant protection. Antibiotic prophylaxis with ceftriaxone, ciprofloxacin, azithromycin, or rifampin (option D) is usually the recommended course of action for high-risk close contacts to reduce nasopharyngeal carriage of the bacteria and prevent secondary disease. Options B and C involve prescribing doxycycline and penicillin respectively, which are generally not used for meningococcal prophylaxis. As for option E, prophylaxis may be warranted given the close contact with a confirmed meningococcal case. (3) CLINICAL REASONING: In this girl's situation, close contact with a diagnosed case of meningitis puts her at a higher risk of infection. While she doesn't currently show symptoms, we need to mitigate the risk of her falling ill, due to the potential severity of meningitis. Since she's already potentially exposed, the best course of action is to reduce the bacteria's ability to colonize and multiply in her body, rather than relying on a vaccine that will take time to create immunity. (4) CONCLUDING: Given these considerations, prophylactic antibiotic therapy is the most appropriate course of action. Among the alternatives, rifampin is typically recommended for contacts of persons with meningococcal disease, and is the most effective agent known for eradicating meningococci from the nasopharynx. (5) RESPONSE: Given the high risk of secondary infection for close contacts of patients with meningococcal disease, the best preventative measure is antibiotic prophylaxis. Thus, the council's recommendation is to prescribe rifampin (Answer D), which is known for its effectiveness in clearing meningococci from the nasopharynx.

**AI_D:** RESTATING THE QUESTION: The situation involves a healthy 16-year-old girl with a history of asthma who is worried she may have been exposed to meningitis. She's in close contact with a teammate recently hospitalized with meningococcal meningitis. Even though she's asymptomatic with normal vital signs, her concerns and close contact with someone infected raise questions about the necessity of preventive measures. The options presented include the meningococcal vaccine, prescribing doxycycline, penicillin, or rifampin, or assuring the patient that no prophylaxis is necessary. Meningitis is an inflammation of the meninges, often caused by bacterial or viral infection. One of the most severe forms is caused by the bacterium Neisseria meningitidis, responsible for meningococcal meningitis. Meningococcal disease is primarily transmitted through droplets of respiratory or throat secretions from carriers. PLANNING: The main question is whether she may have contracted the disease and if she needs specific intervention. Assumptions to consider include the probability of infection from close contact, whether she already possesses immunity, and the most effective prophylactic treatment. Since contraction probability is increased due to the close contact, and it is imprudent to assume existing immunity without vaccination evidence, it seems important to explore the optimal prophylactic response. CLINICAL REASONING: In such potential meningococcal meningitis exposure cases, preventive therapy is typically recommended because this disease can spread easily among those in close contact. We must choose between antibiotics and vaccination. A vaccine is mostly useful as a long-term protective measure, but it does not provide immediate immunity. In an exposure scenario, immediate protection is key. Therefore, we prioritize an antibiotic course, specifically those that can eliminate meningococci from the nasopharynx, where harboring can occur. The three antibiotics commonly used for this purpose are rifampin, ciprofloxacin, and ceftriaxone. Due to her age, ciprofloxacin and ceftriaxone would be avoided. Penicillin and doxycycline are not effective for prophylaxis in the setting of a known exposure. CONCLUDING: Given the need to prevent the potential progression of disease following exposure to meningococcal meningitis and weighing the efficacy of the proposed interventions, the appropriate choice would be the use of an antibiotic known for successfully eliminating nasopharyngeal carriage of Neisseria meningitidis, and safe to use in this age group RESPONSE: (D) Prescribe rifampin. Rifampin is a potent antibiotic against Neisseria meningitidis, known for its ability to eliminate nasopharyngeal carriage of this bacterium without the safety concerns associated with ciprofloxacin and ceftriaxone in younger patients. It should provide the immediate preventative treatment needed following her potential exposure.

**AI_A:** (1) RESTATING THE QUESTION: The inquiry put forth is regarding a 16-year-old girl who, despite lacking any symptoms, fears she may have contracted meningococcal meningitis from a close contact. Her companion, a teammate, was recently diagnosed with this communicable disease, leading to concerns about potential exposure and consequent infection. Given her medical history of asthma and her current health status displaying no evident abnormalities, we are tasked to identify the best course of action. Meningococcal meningitis is a bacterial form of meningitis caused by the bacterium Neisseria meningitidis. It's a serious infection of the meninges that can cause severe brain damage and is often fatal. Close quarters, such as the shared hotel room described, can facilitate the spread of this disease. (2) PLANNING: Several options are laid out including vaccination, different antibiotic prescriptions (doxycycline, penicillin, rifampin) and the option of no intervention at all. The assumptions made in proposing these courses of action are based on methods of either prevention (vaccine), treatment (the specific antibiotics) or the belief of no risk and hence no need for any intervention. Considering the close contact with a confirmed case, a measure of prudence might involve preventative action, particularly when the exposed individual has no symptoms but has potential risk. We however, may question the sufficiency and timing of vaccination in response to potential imminent infection. Meanwhile, the prescribing of antibiotics assumes that the patient will certainly contract the infection and requires immediate treatment, a rather hard assumption to adopt without any symptom manifestation. What merits further investigation, however, is the use of any of these antibiotics as a prophylactic measure, hence preemptive of disease onset. The final assumption questions whether any intervention is necessary considering the absence of symptoms and the very plausible chance that the patient did not contract the disease despite the noted close contact. (3) CLINICAL REASONING: Close contact in confined spaces is a risk factor for transmission of the Neisseria meningitidis bacterium. According to the Centers for Disease Control and Prevention (CDC), prophylactic antibiotics are recommended for anyone who has had close contact with a patient with meningococcal meningitis. Although not yet symptomatic, it takes between 1-10 days after exposure to start showing symptoms. This, coupled with the fact that transmission is easy in crowded settings, justifies a prophylactic approach. While the meningococcal vaccine is useful for preventing meningitis, it is not effective post-exposure since it does not provide immediate protection. It usually takes about two weeks after vaccination to develop immunity. This rules out option A. Moving on to the antibiotics, doxycycline and penicillin are both effective against Neisseria meningitidis, but are not first-line options. According to the CDC, rifampin is the drug of choice for prophylaxis against meningococcal meningitis. Assuring the patient that no prophylaxis is necessary, considering she was directly exposed and in close contact with an infected person, is not an appropriate approach. (4) CONCLUDING: With this step-by-step comparison between the proposed interventions and considering the patient's immediate risk, it becomes clear that providing prophylaxis is justifiable and necessary. (5) RESPONSE: Therefore, the most appropriate intervention would be (D) Prescribe rifampin. This action best reduces the girl's risk of contracting meningococcal meningitis, considering her recent exposure to a confirmed case.

**Facilitator** The consensus among the AI responses is that the most appropriate intervention for the 16-year-old girl, who has been in close contact with a confirmed case of meningococcal meningitis, is to prescribe rifampin (Option D). This conclusion is based on the following synthesized reasoning: 1. Meningococcal meningitis is a serious bacterial infection caused by Neisseria meningitidis. Close contact, such as sharing a hotel room, increases the risk of transmission. Despite the girl currently showing no symptoms, the incubation period for the disease can range from 1-10 days, and her recent exposure to a confirmed case justifies a prophylactic approach. 2. The options presented include vaccination, prescribing various antibiotics (doxycycline, penicillin, rifampin), or assuring the patient that no intervention is necessary. However, the meningococcal vaccine does not provide immediate protection and takes about two weeks to develop immunity, making it unsuitable for post-exposure scenarios. 3. The use of antibiotics such as doxycycline and penicillin is not recommended as first-line options for prophylaxis against meningococcal meningitis. The Centers for Disease Control and Prevention (CDC) recommends rifampin as the drug of choice for prophylaxis in such cases. 4. Assuring the patient that no prophylaxis is necessary, despite her direct exposure and close contact with an infected person, is not an appropriate approach due to the significant risk of her contracting the disease. 5. Therefore, prescribing rifampin is the most suitable intervention. It is a potent antibiotic against Neisseria meningitidis, known for its ability to eliminate nasopharyngeal carriage of this bacterium, providing the immediate preventative treatment needed following potential exposure.
